# Metabolic abnormalities in adolescents with polycystic ovary syndrome in south china

**DOI:** 10.1186/1477-7827-8-142

**Published:** 2010-11-17

**Authors:** Jia Huang, Renmin Ni, Xiaoli Chen, Lili Huang, Yaqin Mo, Dongzi Yang

**Affiliations:** 1 Department of Obstetrics and Gynecology, Memorial Hospital of Sun Yat-Sen University, Guangzhou, Guangdong, China; 2 Department of Obstetrics and Gynecology, The First Affiliated Hospital of Kunming Medical College, Kunming, Yunnan, China

## Abstract

**Background:**

Adults with polycystic ovary syndrome (PCOS) can have multiple metabolic abnormalities. However, studies in the adolescent population are still limited and these results seem to vary widely. This study was to investigate the metabolic abnormalities in adolescents with PCOS in South China and the potential risk factors contributed to these health risks.

**Methods:**

Anthropometric measurements and biochemical parameters were evaluated in 128 adolescents with PCOS and their age- and BMI-matched controls.

**Results:**

The prevalence of pre-diabetes, insulin resistance, hyperinsulinemia, dyslipidemia and metabolic syndrome in adolescents with PCOS was 11.7%, 46.9%, 29.7%, 22.7% and 4.7%, respectively. 16.3%, 74.4%, 67.4%, 39.5% and 14% of the PCOS subjects with BMI > 85th had pre-diabetes, insulin resistance, hyperinsulinemia, dyslipidemia and metabolic syndrome, whereas 9.4%, 32.9%, 10.6%, 14.1% and 0% of the PCOS subjects with BMI < 85th had such disturbances.

**Conclusions:**

Adolescents with PCOS in South China had more metabolic abnormalities than their age- and BMI-matched non-PCOS counterparts. Obesity could worsen insulin resistance, hyperinsulinemia and metabolic syndrome in PCOS adolescents.

## Background

Polycystic ovary syndrome (PCOS) is a heterogeneous endocrine disorder that affects about 6%-8% women worldwide [1]. The consequences of the PCOS extend beyond the reproductive axis; women with the disorder are at substantial risk for developing metabolic abnormalities [2]. Perpetual sequence of hormonal and metabolic aberrations in PCOS patients may commence early, even in adolescent period, and extend throughout life.

Metabolic abnormalities, including pre-diabetes, dyslipidemia and metabolic syndrome (MS), have been widely studied in adult women with PCOS. Women with PCOS tend to have more than two to four times metabolic disturbances compared to those without PCOS [3-5]. However, there are few studies on the metabolic disturbances in adolescents with PCOS all over the world. Moreover, our previous studies also found a lower prevalence of the abnormal glucose tolerance and MS in Chinese women [6,7]. Therefore, we sought to exhibit the metabolic abnormalities in adolescents with PCOS.

40-60% of women with PCOS are overweight or obese. The role of obesity as a contributing factor in the development of PCOS has been widely accepted [8]. Body mass index (BMI) levels correlate with both body fat and concurrent health risks. Hence, we explored the relationship between BMI levels and metabolic abnormalities in Chinese adolescents with PCOS.

The aim of this study was to investigate the characteristics of metabolic abnormalities in adolescents with PCOS in South China, and to explore their potential risks, especially obesity. We sought to determine whether screening adolescents for metabolic abnormalities would benefit.

## Methods

### Subjects and study design

We performed a cross-sectional study of 128 adolescents with PCOS recruited from the gynecological outpatient department of Memorial Hospital of Sun Yat-Sen University from January 2004 to December 2009. PCOS was defined by the Rotterdam 2003 criteria [2]. PCOS subjects should present at least two of the following criteria: oligo- and/or anovulation (i.e. ≤ 8 menstrual periods in a year or menstrual cycles more than 35 days in length); clinical hyperandrogenism (i.e. acne or modified Ferriman-Gallwey scores ≥ 8) and/or biochemical hyperandrogenism (i.e. serum total testosterone(TT) ≥ 2.6 nmol/l, free testosterone (FT) ≥ 6.0 pg/ml, TT and FT normal values were determined by the clinical laboratory of the gynecology department at the Memorial Hospital of Sun Yat-Sen University); and polycystic ovaries (i.e. the presence of ≥ 12 follicles in each ovary measuring 2-9 mm in diameter) after exclusion of other etiologies (e.g. congenital adrenal hyperplasia, androgen-secreting tumours and Cushing's syndrome). The Rotterdam criteria have expanded rather than replacing the NIH criteria, because all women diagnosable by the NIH 1990 criteria would also meet the Rotterdam definition. This study could analyze two subgroups of PCOS according to different diagnostic approach.

Age- and BMI-matched controls were recruited from a total of 654 adolescents visiting the health-care center for an annual routine check-up. They were asked to fill out a questionnaire on their menstrual cycle pattern during last year and received anthropometric measurements. In total, 593 (90.7%) completed, among which 89.4% (530) had normal menstruation (defined by average length of the menstrual cycle between 21-35 days, and menstrual period less than 7 days.), but only less than 100 adolescents participated in further testing. After exclusion of polycystic ovaries by transrectal ultrasound, 40 adolescents served as the age- and BMI-matched controls.

This study was approved by the institutional review board of the Hospital. Informed consent was obtained either from a legal guardian of each subject less than 18 years old or from those subjects 18 years old or older. Any medications known to affect sex hormone, glucose or lipid metabolism were discontinued for at least a month, with the exception of oral contraceptives for 3 months before the study. All subjects did not use any hypolipidemic or anti-hypertensive drugs.

### Study protocols

All subjects who were enrolled in the study had a medical history and underwent physical examination, including weight, height, waist and hip circumferences, blood pressure, modified Ferriman-Gallwey scores, and acne scores. All measurements were done by observers trained with the same standard protocols [9-11]: waist circumference was measured midway between the lowest rib and the iliac crest with the subject standing at the end of gentle expiration, and hip circumference at the greater trochanters; blood pressure was measured twice with mercury sphygmomanometer, with subjects seated quietly for at least 5 minutes, and the readings were averaged as the final value. Ferriman-Gallwey scores were assessed by at least two observers; Transrectal ultrasound examination was performed to evaluate the ovaries using a Toshiba Sonolayer SSA-220A (Toshiba, Tokyo, Japan) with a mechanical 6-MHz probe. Adolescents with regularly menstruation were scanned in the early follicular phase (cycle days 3-5), while those with oligomenorrhea or amenorrhea were scanned either at random or between days 3 and 5 after a progestin-induced withdrawal bleeding.

After a 10-hour overnight fasting, blood samples were taken from adolescents between the first and fifth day of the menstrual period/withdrawal bleeding in order to measure PRL, LH, FSH, estradiol (E_2_), total testosterone (TT), free testosterone (FT), sex hormone-binding globulin (SHBG), DHEAS, 17-OHP, TSH, and lipid profile. An oral glucose-tolerance test (OGTT) using 75 g of glucose was then performed, and blood samples were taken at 0, 60, and 120 min for glucose and insulin measurement. HOMA-IR was calculated as (fasting plasma glucose (mmol/l)*insulin (mU/ml))/22.5.

Impaired fasting glycaemia (IFG), impaired glucose tolerance (IGT) and diabetes mellitus (DM) were defined by the criteria proposed by the American Diabetes Association (ADA) [12]. Patients with IFG and/or IGT are now referred to as pre-diabetes [12]. Dyslipidemia was defined as having one or more of the following: CHOL ≥ 6.0 mmol/L, TG ≥ 1.7 mmol/L, HDL-C < 1.29 mmol/L and LDL-C ≥ 3.6 mmol/L. Insulin resistance (IR) was defined as the HOMA-IR value ≥ 95^th ^percentile, and hyperinsulinemia (HIN) as the fasting insulin level ≥ 95^th ^percentile [13]. MS was defined by International Diabetes Federation (IDF) criteria for MS in children and adolescent [14,15]. Adult IDF criteria also apply to adolescents more than 16 years [15], if they had central obesity (waist circumference ≥ 80 cm) plus two or more of the following four factors: 1) increased concentration of TG ≥ 1.7 mmol/l; 2) reduced concentration of HDL-C < 1.29 mmol/l; 3) raised blood pressure: systolic pressure ≥ 130 mmHg or diastolic pressure ≥ 85 mmHg or treatment of previously diagnosed hypertension; and 4) increased fasting glucose level ≥ 5.6 mmol/l or previously diagnosed type 2 diabetes.

### Assays

PRL, LH, FSH, E_2_, and TT were measured by the chemiluminescence immunoassays Access 2 (Beckman, USA); FT, SHBG, DHEAS, and 17-OHP were measured by Access 2 ELISAs (Beckman, USA). TSH was measured using a chemiluminescence immunometric assay (Immulite 2000 Analyzer; CPC, Los Angeles, CA, USA). Total cholesterol (CHOL), high-density lipoprotein cholesterol (HDL-C), low-density lipoprotein cholesterol (LDL-C) and triglyceride (TG) were measured using an enzymatic calorimetric method with the 7600 autoanalyzer (Hitachi 7600). Plasma glucose was measured using the glucose oxidase method (Hitachi 7600) and plasma insulin using a chemiluminescence immunometric assay and commercial kit (Immulite 2000 Analyzer; CPC).

### Statistical analysis

All data were analyzed using SPSS version 13.0 (SPSS Inc., Chicago, IL, USA). We assessed the normality of the distribution of all continuous variables using the Kolmogorov-Smirnov test. Because the data were not normally distributed, continuous variables were presented as medians and the interquartile ranges. Medians between patients and controls were compared with the Mann-Whitney U test. Pearson χ^2 ^test was used to analyze the differences between the groups and obtain an odds ratio (OR). Statistical significance was set at *p *< 0.05.

## Results

### Demographics and anthropometrics

In total, 128 adolescents were diagnosed as PCOS according to the Rotterdam criteria. Of these, 96.1% presented oligomenorrhea or amenorrhea, all persisting for 2 years post menarche; 83.6% presented ultrasonographic appearance of polycystic ovaries; 74.2% presented clinical and/or biochemical hyperandrogenism. In total, 70.3% (90/128) of patients had both androgen excess and ovulatory dysfunction and met the NIH 1990 criteria.

The comparison of demographic characteristics between adolescents with PCOS and controls was summarized (Table 1). No differences were found between the controls whether had given a blood sample or not, concerning age, BMI, duration of the period, and quantity of menstrual flow. There were no differences in age, menarche age, gynecological age, and BMI between the subjects with and without PCOS. Subjects with PCOS had increased waist circumference, higher FG scores and blood pressure. The subjects with PCOS defined by different criteria presented almost the same results when compared with controls, except for the waist/hip ratio.

**Table 1:** Demographic characteristics of adolescents with and without PCOS

		PCOS	
	Control	Rotterdam criteria	NIH criteria
Number of subjects	40	128	90
Age(yr)	19.0 (17.0-19.0)	18.0 (17.0-19.0)	18.0 (17.0-19.0)
Menarche(year)	12.5 (12.0-13.0)	12.0 (12.0-13.0)	12.0 (12.0-13.0)
Gynecological age(year)	6.0 (4.0-7.0)	6.0 (5.0-7.0)	6.0 (5.0-7.0)
BMI(kg/m2)	19.6 (18.4-21.0)	20.0 (18.5-25.1)	20.2 (18.4-25.2)
Waist(cm)	67.3 (63.0-72.8)	72.0 (67.5-80.0) **	72.0 (68.0-80.4) **
WHR	0.78 (0.75-0.81)	0.80(0.76-0.84)	0.80(0.76-0.85) *
SBP(mmHg)	100.0 (92-105)	110.0 (100-120) **	110.0 (98-120) **
DBP(mmHg)	60.0 (60-66)	71.0 (65-78) **	71.0 (65-78) **
FG scores	0 (0-5.5)	9 (6-13) **	10 (7-13) **

Variables were expressed as median with (interquartile range).

Gynecological age = calendar age - menarche age; BMI: body mass index; WHR: waist to hip ratio; SBP: systolic pressure; DBP: diastolic pressure; FG scores: Ferriman-Gallwey scores.

*P < 0.05, **P < 0.01 vs. control.

Table 2 listed the hormonal and metabolic features of adolescents with or without PCOS. Subjects with PCOS displayed significantly higher LH, TT, FT, 2-h glucose, fasting insulin, 2-h insulin, and HOMA-IR, but there were no significant differences in FSH, fasting glucose, TG, CHOL, LDL and HDL between the PCOS and the control groups.

**Table 2:** Hormonal and metabolic features of adolescents with and without PCOS

		PCOS	
	Control	Rotterdam criteria	NIH criteria
FSH(IU/L)	5.3(4.4-6.6)	5.6(4.5-6.9)	5.6(4.5-6.9)
LH(IU/L)	5.1(3.6-7.5)	11.0(5.9-16.1) **	10.3(6.3-16.2) **
TT(nmol/L)	1.7(1.5-2.4)	2.5(2.0-3.2) **	2.8(2.3-3.3) **
FT(pg/ml)	2.9(2.1-3.5)	4.6(2.6-7.3) **	5.1(2.9-8.1) **
Fasting glucose(mmol/L)	4.7(4.5-4.8)	4.8(4.5-5.0)	4.8(4.5-5.1)
2-h glucose(mmol/L)	5.6(5.0-6.3)	6.3(5.1-7.1) *	6.3(5.3-7.1) **
Fasting insulin(μU/ml)	5.7(4.4-8.3)	8.8(5.7-13.2) **	9.0(5.9-13.2) **
2-h insulin(μU/ml)	48.1(35.5-82.4)	72.7(46.6-131.9) *	76.7(51.0-139.) **
HOMA-IR	1.2(0.9-1.8)	1.9(1.2-2.9) **	1.9(1.4-2.9) **
CHOL(mmol/L)	4.2(3.7-4.7)	4.5(4.1-5.1)	4.5(4.1-5.1)
TG(mmol/L)	0.9(0.7-1.2)	1.0(0.8-1.3)	1.0(0.7-1.3)
HDL(mmol/L)	1.6(1.4-1.7)	1.6(1.4-1.9)	1.5(1.3-1.9)
LDL(mmol/L)	2.6(2.1-2.9)	2.5(2.1-3.1)	2.5(2.1-3.2)

Variables were expressed as median with (interquartile range).

FSH: follicle stimulating hormone; LH: luteinizing hormone; TT: total testosterone; FT: free testosterone; HOMA-IR: Homeostasis Model Assessment of Insulin Resistance; CHOL: total cholesterol; TG: triglyceride; HDL: high-density lipoprotein cholesterol; LDL: low-density lipoprotein cholesterol.

*P < 0.05, **P < 0.01 vs. control.

### Prevalence of metabolic abnormalities in adolescents with and without PCOS

The prevalence of various metabolic abnormalities in adolescents with and without PCOS was listed in Table 3. No DM was detected either in the PCOS group or in the control group. Pre-diabetes (IFG and/or IGT) was only found in the PCOS group, and the prevalence of IFG, IGT and pre-diabetes was 1.6%, 10.2% and 11.7%, respectively. The prevalence of hyperinsulinemia and insulin resistance raised more than 2.5 folds in PCOS group (29.7% vs. 7.5%; 46.9% vs.17.5%). PCOS group also presented higher prevalence of central obese (20.3% vs. 5%). However, there were no significant differences in lipid disturbances and other components of MS between groups. The characteristics were consistent, no matter which diagnostic criteria were used to define PCOS.

**Table 3:** Prevalence of various metabolic abnormalities in adolescents with and without PCOS

		PCOS (%)	
	Control (%)	Rotterdam criteria	NIH criteria
IFG	0	1.6	2.2
IGT	0	10.2*	12.2*
Pre-diabetes	0	11.7*	14.4**
HIN	7.5	29.7**	33.3**
IR	17.5	46.9**	44.4**
Hypercholesterolemia	0	4.7	5.6
Elevated LDL	0	4.7	5.6
Low HDL	15.0	14.1	16.7
Hypertriglyceridemia	7.5	10.2	12.2
Dyslipidemia	17.5	22.7	26.7
Central obese	5.0	20.3*	24.4**
Raised BP	2.5	8.0	8.2
MS	2.5	4.7	6.7

IFG: impaired fasting glycaemia; IGT: impaired glucose tolerance; pre-diabetes: IFG and/or IGT; HIN: hyperinsulinemia; IR: insulin resistance; MS: metabolic syndrome.

*P < 0.05, **P < 0.01 vs. control.

### Prevalence of metabolism abnormalities in different BMI subgroups of adolescents with PCOS

Of the sample of 530 otherwise healthy adolescents, the 85^th ^and 95^th ^percentile of BMI was 21.4 kg/m^2 ^and 23.8 kg/m^2^, respectively. 33.6% (43/128) subjects with PCOS by Rotterdam criteria had a higher BMI above the 85^th ^percentile, and 36.7% (33/90) subjects by NIH criteria.

**Figure 1:**
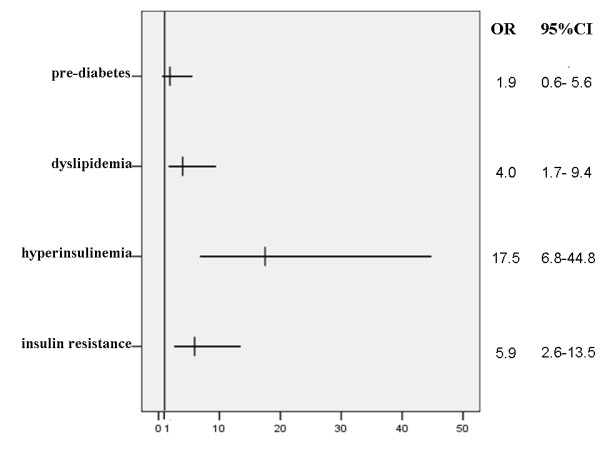
**Odds ratio for four clinical disorders in BMI ≥85th versus BMI < 85th groups of adolescents with PCOS**. PCOS was defined by Rotterdam criteria.

To assess the effect of BMI on the prevalence of pre-diabetes, dyslipidemia and metabolic syndrome, the subjects with PCOS were then divided into two groups, group BMI ≥ 85^th ^and group BMI < 85^th^. Although there was no statistical significance in pre-diabetes and hypercholesterolemia between the two groups (Table 4), group BMI ≥ 85^th ^had higher risk of hyperinsulinemia (67.4% vs. 10.6%; OR, 17.5; 95%CI, 6.8-44.8), insulin resistance (74.4% vs. 32.9%; OR, 5.9; 95%CI, 2.6-13.5), and dyslipidemia (39.5% vs. 14.1%; OR, 4.0; 95%CI, 1.7-9.4) than those of group BMI < 85^th ^(Figure 1). Moreover, metabolic syndrome was only detected in group BMI ≥ 85^th^.

**Table 4:** Prevalence of metabolic abnormalities in adolescents with PCOS categorized by different BMI

	Rotterdam criteria		NIH criteria	
BMI	< 85th	≥ 85th	< 85th	≥ 85th
Number of subjects	85	43	57	33
IFG	2.4	0	3.5	0
IGT	7.1	16.3	10.5	15.2
pre-diabetes	9.4	16.3	14.0	15.2
HIN	10.6	67.4 ***	12.3	69.7 ***
IR	32.9	74.4 ***	28.1	72.7 ***
Hypercholesterolemia	2.4	9.3	3.5	9.1
Elevated LDL	1.2	11.6*	1.8	12.1
Low HDL	8.2	25.6**	10.5	27.3 *
Hypertriglyceridemia	3.5	23.3 **	3.5	27.3**
Dyslipidemia	14.1	39.5**	17.5	42.4 *
Central obese	0	60.5 ***	0	66.7 ***
Raised BP	0	19.4**	0	18.5 *
MS	0	14.0 **	0	18.2**

Abbreviations as in Table 3.

*P < 0.05, **P < 0.01, ***P < 0.001 vs. the "BMI < 85th" value.

## Discussion

PCOS can have multiple metabolic disturbances, such as insulin resistance and compensatory hyperinsulinemia, impaired glucose tolerance, dyslipidemia and metabolic syndrome [16]. Concerning the long-term health risk, it is necessary to identify such metabolic disorders in those adolescents with PCOS as early as possible. However, data on metabolic disturbances in adolescents with PCOS are limited, and the results seemed to vary in studies of different populations. In this study, we first outlined the metabolic disturbances in Chinese adolescents with PCOS, and then we also studied the potential risk factors in such population.

Our data showed that the prevalence of IFG and IGT was 1.6% and 10.2%, respectively. No T2DM was detected. A Canadian study of 22 obese adolescents with PCOS showed only one patient (4.5%) suffered from IGT [17]. In contrast, two American studies from a similar geographic background population both indicated a higher prevalence of IGT in obese adolescents with PCOS (29.6%, 8 in 27 [18]; 27.3%, 3 in 11 [19], respectively). Considering the confounding effects of ethnicity and geographical background on the prevalence of metabolic disturbances, we also searched literatures published by Asian researchers, and only found one Korean study in which 1 in 16 (6.3%) adolescents with PCOS had IGT [20]. In fact, the variations among these studies may due to the small sample size of subjects. However, a study report of 101 overweight Hispanic adolescents revealed that the prevalence of IGT and DM was 6.6% and 2%, respectively [21]. In our study, the prevalence of IGT among overweight (BMI ≥ 85^th ^according to pediatric criteria [22]) subjects with PCOS was 16.3%. Considering more detail of the different study population, the variation may due to the elder age in our study population.

Data from Nur et al [21] and our study indicated adolescents with PCOS had a lower prevalence of either pre-diabetes or T2DM than their same ethnicity and geographical background adults. The prevalence of IGT was 31-35% in adults, while DM was 7.5-10% [23,24]. Although in our previous study the prevalence in Chinese adults with PCOS was lower (IGT 20.5%, T2DM1.9%) than that of American PCOS [6], the prevalence was still higher than that of Chinese adolescents. Given that the conversion from IGT to T2DM is substantially enhanced in women with PCOS [25,26], the early identification of affected patients and institution of lifestyle changes or drug treatments may benefit. Like other studies, we noted that the fasting glucose failed to predict the majority of the subjects with IGT, confirming the recommendation that the most reliable screening test for IGT in PCOS adolescents is the 2-h OGTT after a 75 g glucose load [3].

The prevalence of MS, diagnosed by IDF criteria, was 4.7% in the studied Chinese adolescents with PCOS. Metabolic disorders were common in these adolescents as more than one third of them exhibited at least one component of MS. Central obesity and dyslipidemia were the two most common metabolic features, which was also the case in adult PCOS patients according to our previous study [7]. A study of 49 white of non-Hispanic adolescents with PCOS revealed the prevalence of MS was 37%; the mean BMI of such population was 32 kg/m^2 ^[27]. In India, 17 in 39 (43.6%) subjects with mean BMI 29.3 kg/m^2 ^suffered from MS [28]. While the subjects with BMI 25 kg/m^2^, the prevalence reported in Italy was 9.4% (5 in 53) [29]. Taken together all these results evidence that the MS can be found in PCOS women as early as in adolescence, however, the exact prevalence is not sure. The difference may due to several factors. First, there are no consistent criteria for the diagnosis of MS in adolescents. Important demographic and clinical differences exist in the typology of MS, depending on the definition [30-32]. Second, the ranges of age in these studies are quite different. Age is regarded as an important risk factor for MS in both the general population and PCOS patients. Data of our previous study indicated the increasing trend in the prevalence of MS [7]. Moreover, age could affect the diagnosis of PCOS, because of the physiologic irregular ovulation, insulin resistance, and multi-follicular ovarian morphology characteristic of normal adolescents. Thus, the diagnosis of PCOS in young adolescents should be prudent [33,34]. Third, obesity is an early step in the etiological cascade leading to full metabolic syndrome. Increased body weight in female significantly increases the risk of having MS [35]. Difference BMI levels could account for variations in the prevalence of MS. Other factors, such as sample size, genetic factors, dietary, exercise and geographic location may also have some effects.

In this study, the characteristics of metabolic disturbances also exhibited according to different diagnosis criteria for PCOS. Specialized criteria for the diagnosis of PCOS in adolescents have not been established yet, and instead, the diagnostic criteria for adults are used in clinical practice. At present, the 2003 Rotterdam criteria are more widely accepted than the NIH criteria, but the former tends to expand rather than replace the latter because all women diagnosed by the NIH 1990 criteria would also meet the Rotterdam criteria. The risk of metabolic disturbances may vary among different phenotypes of PCOS based on the Rotterdam criteria. Adults PCOS defined by NIH criteria had more severe metabolic abnormalities than those defined by Rotterdam criteria. The difference of prevalence of obesity and androgen excess may attribute to the characteristic [36-38]. In an attempt to avoid erroneous diagnoses, we decided to enroll female adolescents with more than two gynecologic years because a normalization of menstrual cycles mainly occurs within the first 2 years after menarche [39,40]. In our group, the median gynecologic age was 6 years. There were no significant differences in metabolic abnormities between the two groups categorized by two different criteria. In total, 70.3% (90 in 128) subjects met both the criteria. The number of non-NIH subjects group was small, which may weaken the power to detect the differences of metabolic features. Moreover, there were similar levels of BMI, FG score, TT and FT between the Rotterdam group and NIH group. Considering the effects of BMI and androgen on metabolic abnormities, it was not surprising to fail to detected the differences in the two groups PCOS.

Nearly 50% of PCOS patients suffer from obesity, although it is not included in the diagnostic criteria. Obesity contributes to the manifestations of PCOS by increasing the magnitude of hyperandrogenism and the rates of anovulatory cycles and infertility. The pathophysiologic mechanism is related to hyperinsulinemia which, in turn, is induced by insulin resistance [41]. Although many patients with PCOS had insulin resistance independent of obesity [42,43], the obesity indeed worsen underlying insulin resistance and insulin resistance-associated reproductive and metabolic features. Our study suggested that, when matched for obesity, PCOS was associated with increased risks of insulin resistance, hyperinsulinemia and pre-diabetes, but was not associated with an increased risk of MS. Further analysis of PCOS subjects revealed that obesity was associated with increased risks of insulin resistance, hyperinsulinemia and MS, but was not associated with pre-diabetes. The PCOS status and obesity are both associated with the higher prevalence of insulin resistance and hyperinsulinemia. As a central pathogenetic feature of PCOS, insulin resistance could be worsened by obesity. And the impact of obesity is a more significant contributor to the prevalence of MS than PCOS status. In a word, our study emphasized the importance of PCOS status and obesity in adolescents.

### Study limitation

Because of the cross-sectional design and modest sample size of the present study, we could not be certain whether these markers showed ethnic variation in their relation to clinical events such as MS. This would require a much larger prospective study of metabolic disorders in each of several ethnic groups. And due to the difficulties in inviting healthy adolescents to participate in the study, especially scaring of the transrectal ultrasound, uncomfortable and time-consuming OGTT test, there were limit controls fulfill all test.

## Conclusions

Adolescents with PCOS in South China had more metabolic abnormalities than their age- and BMI-matched non-PCOS counterparts. Obesity could worsen insulin resistance, hyperinsulinemia and metabolic syndrome in PCOS adolescents.

## Competing interests

The authors declare that they have no competing interests.

## Authors' contributions

J.H. performed the study, analyzed and interpreted the data and drafted the paper. R.N. and X.C. performed collected, analyzed the data and revised the draft. L.H. and Y.M. performed guided analysis data and performed the blood sample analysis. D.Y. designed, performed the study and revised the paper. All authors read and approved the final manuscript.
